# The Ferumoxytol for Anemia of CKD Trial (FACT)—a randomized controlled trial of repeated doses of ferumoxytol or iron sucrose in patients on hemodialysis: background and rationale

**DOI:** 10.1186/s12882-017-0523-8

**Published:** 2017-04-03

**Authors:** Iain C. Macdougall, Naomi V. Dahl, Kristine Bernard, Zhu Li, Alka Batyky, William E. Strauss

**Affiliations:** 1 Department of Renal Medicine, King’s College Hospital, Denmark Hill, London UK; 2 AMAG Pharmaceuticals, Inc., 1100 Winter Street, Waltham, MA 02451 USA

**Keywords:** Chronic kidney disease, Ferumoxytol, Hemodialysis, Iron overload, Iron sucrose, Oxidative stress

## Abstract

**Background:**

Iron deficiency anemia (IDA) is a common manifestation of chronic kidney disease (CKD), affecting most patients on hemodialysis and imposing a substantial clinical burden. Treatment with iron supplementation increases hemoglobin levels and can reduce the severity of anemia in patients with CKD. While correcting anemia in these patients is an important therapeutic goal, there is a lack of long-term trials directly comparing intravenous iron therapies in patients with CKD receiving hemodialysis.

**Methods/Design:**

The Ferumoxytol for Anemia of CKD Trial (FACT) is a 13-month, open-label, randomized, multicenter, international, prospective study with 2 substudies. Entry criteria for the main study include adults with IDA (defined as hemoglobin <11.5 g/dL [<115.0 g/L] and a transferrin saturation <30%), serum ferritin <800 ng/mL (<1798 pmol/L), and receiving hemodialysis for ≥3 months. Patients are randomized to receive ferumoxytol (1.02 g over 2 doses) or iron sucrose (1.0 g over 10 doses) during the initial 5-week treatment period. Those with persistent/recurrent IDA over the 11-month observation period will receive additional 5-week treatment periods, as appropriate. The primary efficacy endpoint of the main study is the mean change in hemoglobin from Baseline to Week 5 for each treatment period. The secondary efficacy endpoints include the mean change in transferrin saturation from Baseline to Week 5 and the proportion of patients with a hemoglobin increase of ≥1.0 g/dL at any time from Baseline to Week 5. Safety will be assessed through an examination of the adverse event profile over the course of the study. An “oxidative stress” substudy in approximately 100 patients will assess the effects of treatment on biomarkers of oxidative stress/inflammation during the initial 5-week treatment period, and a magnetic resonance imaging substudy in approximately 70 patients will assess the potential for iron deposition in target tissues over 24 months.

**Discussion:**

FACT fulfills the need for a long-term comparative trial in patients with IDA and CKD receiving hemodialysis. The efficacy and safety results will provide useful information for guiding therapy in this population. Two hundred ninety-six patients have been enrolled, and completion of the main study is expected soon.

**Trial registration:**

ClinicalTrials.gov identifier: NCT01227616 (registered October 22, 2010); EudraCT number: 2010-022133-28

## Background

### Iron deficiency anemia in patients with renal disease: clinical challenges

Iron deficiency anemia (IDA) develops early in the course of renal disease [1] and its prevalence increases with advancing chronic kidney disease (CKD), affecting most patients who require hemodialysis [2, 3]. Iron deficiency in these patients is caused by a variety of factors including low dietary intake of iron due to poor appetite or a low protein diet, chronic iron loss from intestinal bleeding resulting from uremic platelet dysfunction, and increased hepcidin synthesis induced by a chronic inflammatory state that interferes with iron uptake and transfer [4]. While the primary effect of iron deficiency is the development of anemia and its related consequences (e.g., fatigue, decreased exercise capacity, and reduced quality of life), iron deficiency is also associated with other adverse effects, including cardiac dysfunction [5, 6] and exacerbation of restless legs syndrome [7], possibly related to impairments in the generation of cellular energy and oxygen storage in myoglobin in heart and skeletal muscle [2, 4, 8]. Thus, correction of iron deficiency is a critical component of medical management in patients with CKD.

Because of hepcidin upregulation, iron from the diet and oral iron supplements are poorly absorbed in the gut [4]. In addition, oral iron supplements are often associated with gastrointestinal intolerance that may reduce patient adherence with therapy [4]. Furthermore, patients receiving hemodialysis have ongoing and recurrent blood loss associated with the hemodialysis process. As orally administered iron cannot sufficiently replenish the iron deficiency occurring as a direct consequence of hemodialysis-related blood loss and the increased iron demand secondary to the use of an erythropoiesis-stimulating agent (ESA), intravenous (IV) iron has become the standard of care in the population of patients with CKD on hemodialysis. However, there are few randomized controlled trials directly comparing different IV iron formulations [9].

### Study rationale and objectives

Randomized controlled trials of IV iron in patients with CKD receiving hemodialysis are few and are either small (<60 patients) or short term (<3 months). A PubMed literature search was performed using the key search terms “chronic kidney disease” and “IV iron” to obtain all relevant publications of randomized controlled trials of IV iron in patients with CKD on hemodialysis. The search was limited to English-language articles and randomized controlled trials in humans. Abstracts were then manually filtered to obtain only studies with a population of patients with CKD on hemodialysis and an IV iron comparator (including alternate methods of dosing/administration) while eliminating all studies with oral iron and placebo comparators. Table 1 summarizes randomized controlled trials with an IV comparator in which an anemia-related endpoint is included. Given the lack of long-term comparative data (i.e., 6 months being the longest duration, with most studies ≤3 months) between IV iron products, there is a need for extended trials directly comparing IV iron therapies in patients with IDA and CKD receiving hemodialysis so that treatment decisions are more informed. Iron overload is a potential concern of long-term IV iron therapy and consequently is of particular concern for patients on dialysis because of their ongoing need for repetitive dosing over an extended period of time [10, 11], thus there is a need for more long-term safety data on the potential for iron deposition in cardiac, hepatic, and pancreatic tissues, and the impact on liver and thyroid functioning and other metabolic markers. In addition, data suggest that IV iron may be implicated in the induction of oxidative stress/inflammatory markers in patients with CKD and IDA including those on dialysis [12–16].

**Table 1:** Summary of IV iron studies in patients with iron deficiency anemia and CKD receiving HD

Study^a, b^	Patients	N	HD patients, n	Treatments	Duration	Primary endpoint
Active comparator						
Anirban et al. 2008 [24]	CKD-HD, CKD-CAPD, and CKD-NDD	339	210	IV iron dextran IV sodium ferric gluconate complex IV iron sucrose	48 h	Incidence of serious/nonserious adverse drug events
Besarab et al. 2000 [25]	CKD-HD	42	42	IV iron dextran (patients assigned to 1 of 2 dosing regimens)	6 months	Determine if maintenance iron protocol that increased TSAT levels from 20–30% to 30–50% increased erythropoiesis and reduced rHuEPO doses needed to maintain Hb of 9.5–12.0 g/dL (95.0–120.0 g/L) vs. protocol targeting TSAT levels of 20–30%
Charytan et al. 2013 [26]	CKD-HD and CKD-NDD	254	50	IV ferric carboxymaltose Oral, IV, or no iron (standard medical care)	30 days	Safety of the maximum administered dose of ferric carboxymaltose vs. standard medical care
Goldstein et al. 2013 [27]	CKD-HD, CKD-NDD, or CKD-PD	145	91	IV iron sucrose (patients assigned to 1 of 3 doses by weight)	3 months	Composite of Hb ≥10.5–14.0 g/dL (≥105.0–140.0 g/L), inclusive; TSAT ≥20–50%, inclusive; and stable ESA dosing (± 25% of baseline dose)
Kosch et al. 2001 [28]	CKD-HD	59	59	IV ferric gluconate (Ferrlecit) IV iron sucrose	6 months	Differences in Hb, TSAT, ferritin, % hypochromic red blood cells, and rHuEpo dose requirements
Macdougall et al. 2014 [17]	CKD-HD and CKD-NDD	162	70	IV ferumoxytol IV iron sucrose	7 weeks	Descriptive review of adverse effects; change in Hb from Baseline to Day 35
Sav et al. 2007 [29]	CKD-HD, CKD-CAPD, and CKD-NDD	60	60	IV iron dextran IV iron sucrose	48 hourss	Adverse reactions immediately (i.e., within 30 min) or up to 48 h after infusion
Sheashaa et al. 2005 [30]	CKD-HD	48	48	IV iron saccharate complex IV sodium ferric gluconate complex	6 months	Change in serum iron, serum ferritin, TSAT, Hb, and hematocrit
Warady et al. 2005 [31]	CKD-HD Pediatric	66	66	IV sodium ferric gluconate complex 1.5 mg/kg IV sodium ferric gluconate complex 3.0 mg/kg	8-dose treatment with each dialysis + 4 weeks	Mean changes in Hb, hematocrit, TSAT, serum ferritin, and reticulocyte Hb content values from Baseline to 2 weeks after dosing cessation

*CAPD* continuous ambulatory peritoneal dialysis, *CKD* chronic kidney disease, *ESA* erythropoiesis-stimulating agent, *Hb* hemoglobin, *HD* hemodialysis, *IV* intravenous, *NDD* non-dialysis dependent, *PD* peritoneal dialysis, *rHuEPO* recombinant human erythropoietin, *TSAT* transferrin saturation

^a^All studies were randomized controlled trials; ^b^All studies reported adverse events

Comparative data evaluating the efficacy of ferumoxytol versus other IV iron preparations for improving hemoglobin levels in patients with CKD on hemodialysis is limited to a single short-term trial versus iron sucrose (in a study in which approximately 43% were hemodialysis patients with CKD and 57% were non-hemodialysis patients with CKD) [17]. The Ferumoxytol for Anemia of CKD Trial (FACT) study is a 13-month, open-label, randomized, multicenter, international, prospective head-to-head study of 2 different formulations of IV iron (ferumoxytol vs. iron sucrose) in approximately 300 patients with CKD on hemodialysis. The purpose of this study is to gain a better understanding of the long-term safety, efficacy, and frequency of IV iron use in the treatment of IDA in hemodialysis patients with CKD. This study is the first long-term head-to-head randomized controlled trial of IV iron therapy in this patient population. It should be acknowledged that the criteria for IDA utilized in this study, while consistent with current practice guidelines for patients with CKD, may not be standard for other patient populations [18]. The oxidative stress and magnetic resonance imaging (MRI) substudies of this trial will address 2 of the safety issues identified as potential concerns with the long-term use of IV iron therapy.

## Methods/Design

### Main study

**Fig. 1:**
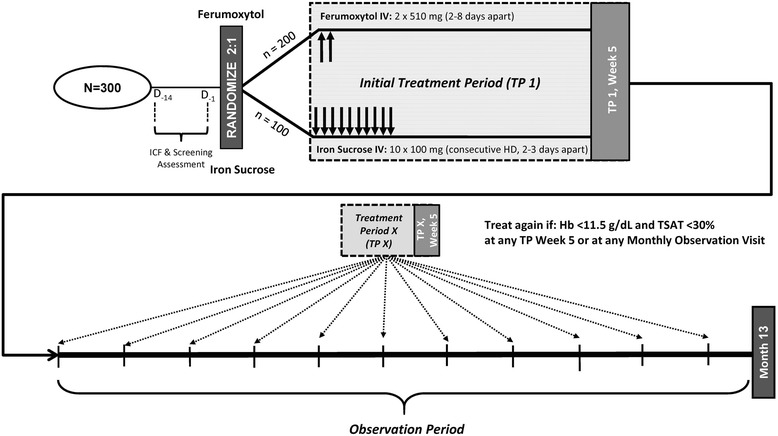
Study design and treatment in main study. *HD* hemodialysis, *Hb* hemoglobin, *ICF* informed consent form, *IV* intravenous, *MRI* magnetic resonance imaging, *TP* treatment period, *TSAT* transferrin saturation

FACT is a phase IV, 13-month, open-label, randomized, multicenter, international, prospective study in approximately 300 adults with CKD on hemodialysis from 35 sites in 3 countries (Fig. 1). At the time of the writing of this manuscript, recruitment has been completed while patients continue to be followed and treated. The following describes the design of the study. The main study will be approximately 13 months in duration, which consists of a 2-week screening period, an initial treatment period of 5 weeks, an 11-month observation period that includes subsequent 5-week treatment periods (whenever treatment criteria are met), and a final visit. The study includes adult patients aged ≥18 years with hemoglobin <11.5 g/dL (<115.0 g/L), transferrin saturation (TSAT) <30%, and serum ferritin <800 ng/mL (<1798 pmol/L). Other inclusion/exclusion criteria are summarized in Table 2. The study is being conducted according to international standards of Good Clinical Practice, the International Conference on Harmonisation, and US Food and Drug Administration regulations, and the protocol was submitted to and approved by the appropriate investigational review boards. All patients provided written informed consent describing the main study and informed consent for each of the substudies, if applicable.

**Table 2:** Key inclusion and exclusion criteria in the Ferumoxytol for Anemia of CKD Trial

Inclusion criteria	Exclusion criteria
Main study	
•Males and females aged ≥18 years	•History of allergy to either oral or intravenous iron
•Patients with iron deficiency anemia defined as hemoglobin <11.5 g/dL (<115.0 g/L) and transferrin saturation <30%	•Female patients who are pregnant or intend to become pregnant, breastfeeding, within 3 months postpartum, or have a positive serum or urine pregnancy test
•Serum ferritin <800 ng/mL (<1798 pmol/L)	•Parenteral iron therapy within 30 days prior to screening or red blood cell/whole blood transfusion within 14 days prior to screening or planned during the study
•Patients must have been on hemodialysis for ≥3 months prior to screening	•Untreated vitamin B_12_ or folate deficiency
•Female patients of childbearing potential who are sexually active must be on an effective method of birth control for ≥1 month prior to screening and agree to remain on birth control until study completion	•ESA therapy initiated, stopped, or dose changed by >20% within 30 days prior to screening, or an anticipated ESA dose change of >20% during the initial treatment period
•Patient is capable of understanding and complying with the protocol requirements and available for the study duration	•Received an investigational agent within 30 days prior to screening or planned receipt of an investigational agent not specified by this protocol during the study period
•Provide written informed consent	•Any other clinically significant medical disease or psychiatric disease or condition that, in the investigator’s opinion, may interfere with the patient’s ability to give informed consent or adhere to the protocol, interfere with assessment of the study agent, or serve as a contraindication to the patient’s participation in the study
MRI substudy	
•Same as main study	•Same as main study •Has any contraindication to MRI or otherwise unable to undergo MRI (e.g., pacemaker, recent wound clips, severe claustrophobia, or unable to lay flat for sufficient time to undergo imaging)
	•Baseline cardiac T2*-weighted MRI value <20 msec

*CKD* chronic kidney disease, *ESA* erythropoiesis-stimulating agent, *MRI* magnetic resonance imaging

### Oxidative stress substudy

**Fig. 2:**
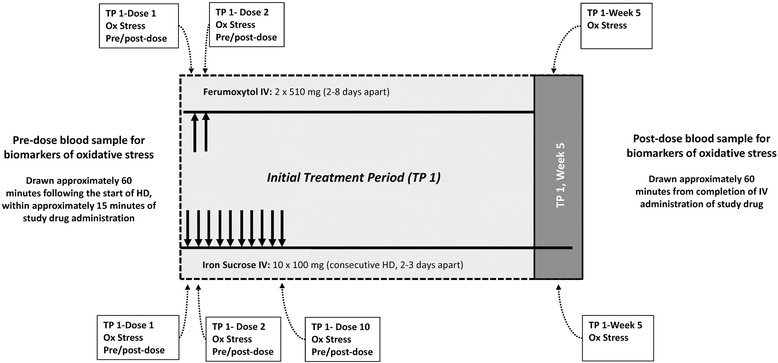
Study design and treatment in oxidative stress substudy. *HD* hemodialysis, *Hb* hemoglobin, *ICF* informed consent form, *IV* intravenous, *MRI* magnetic resonance imaging, *TP* treatment period, *TSAT* transferrin saturation

Approximately 100 patients who enroll in the main study (~50 patients from each treatment group) will participate in an “oxidative stress” substudy, with a participation period of 5 weeks that will run concurrently with the initial treatment period of the main study. Participation in the substudy is optional. Patients who participate in the oxidative stress substudy will only be selected from US-based sites given the challenge of transporting serum to the central laboratory in the US for analysis. No further selection process will be applied. Key time points in the oxidative stress study are summarized in Fig. 2.

### MRI substudy

**Fig. 3:**
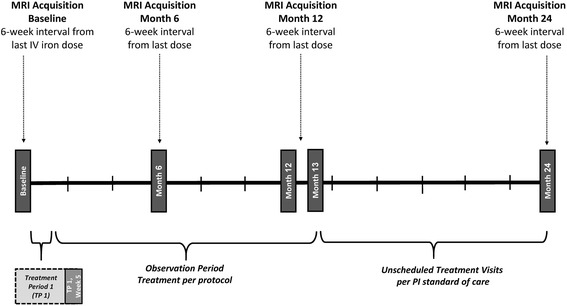
Study design and treatment in MRI substudy. *IV* intravenous, *PI* prescribing information, *TP* treatment period

Approximately 70 patients from the main study (~35 patients from both treatment groups) will participate in the MRI substudy. The option to participate in the MRI substudy will be given to those patients from clinical sites within a reasonable travel distance to one of the specialized MRI sites that had participated in specific training and performance validation of the T2*-weighted MRI (MRI T2*) technique. Additional exclusion criteria for the MRI substudy include: any contraindication to MRI; inability to undergo MRI (e.g., pacemaker, recent wound clips, severe claustrophobia, and unable to lay flat for sufficient time to undergo imaging); and baseline cardiac T2* value <20 ms. After completion of the main study, participating patients will continue in the MRI substudy for approximately 11 additional months (2 years in total) with MRI data acquisition at Baseline and at 6, 12, and 24 months. Key time points in the MRI substudy are summarized in Fig. 3.

### Study objectives

The primary objective is to demonstrate that 1.02-g courses of ferumoxytol (delivered as either 2 undiluted IV injections or diluted IV infusions of 510 mg each) are noninferior to 1.0-g courses of iron sucrose (delivered as either 10 slow, undiluted IV injections or diluted IV infusions of 100 mg each) in increasing hemoglobin after each treatment period in hemodialysis patients with CKD and IDA over 13 months. The safety objective is to evaluate the safety of repeat doses of ferumoxytol compared to iron sucrose for the treatment of IDA over a 13-month period in patients with CKD receiving hemodialysis. The “oxidative stress” substudy will evaluate changes in blood biomarkers of oxidative stress/inflammation during the initial treatment period of the main study. The MRI substudy will assess the potential for iron deposition in parenchymal tissues (heart, liver, and pancreas) and evaluate hepatic, thyroid, and metabolic laboratory parameters over a 2-year period (13-month main study plus 11-month continuation).

### Randomization and interventions

After a screening period of up to 2 weeks, patients will be randomized in a 2:1 ratio to receive either IV ferumoxytol or IV iron sucrose during an initial 5-week treatment period (Fig. 1). An interactive voice or web system will be utilized for randomization and assignment to treatment groups. Ferumoxytol is administered as either an undiluted IV injection or diluted IV infusion of 510 mg with a second 510-mg dose administered 5 (±3) days after the initial dose for a cumulative dose of 1.02 g. Those assigned to the iron sucrose group will receive either slow undiluted IV injection or diluted IV infusion of 100 mg on day 1 and at the following 9 consecutive hemodialysis sessions over approximately 3 weeks for a cumulative dose of 1.0 g. During the 11-month observation period, patients found to have persistent/recurrent IDA (hemoglobin <11.5 g/dL [<115.0 g/L] and TSAT <30%) at any monthly observation visit will receive additional 5-week treatment periods with ferumoxytol or iron sucrose, according to treatment assignment. Iron sucrose was chosen as the comparator as it is the most commonly used IV iron therapy worldwide, is effective, and has a well-characterized tolerability profile. For participants in the main study who are receiving ESA therapy, the ESA dose will not be modified during any 5-week treatment period following administration of ferumoxytol or iron sucrose unless required for patient safety.

### Assessments

#### Laboratory tests

In the main study, blood samples for laboratory tests will be taken prior to study drug administration, at the end of the initial 5-week treatment period, during each observation visit, and at the final visit. For those in the MRI substudy, additional blood samples will be obtained at the final visit (i.e., approximately 24 months from the initial treatment period visit). Laboratory evaluations will include hematology parameters, iron panel, and clinical chemistry.

#### Biomarkers

Blood samples for determination of biomarkers assessed in the “oxidative stress” substudy will be collected for patients randomized to ferumoxytol before and after each of the 2 doses and at Week 5 in the initial treatment period. For those in the iron sucrose group, samples will be collected before and after the 1st, 2nd, and 10th doses and at Week 5 during the initial treatment period. Biomarkers to be assessed include protein carbonyl content, 13-hydroxyoctadecadienoic acid, 4-hydroxynonenal, monocyte chemoattractant protein-1, neutrophil gelatinase-associated lipocalin, and high-sensitivity interleukin-6. Assays of biomarkers will be conducted at a central laboratory.

#### MRI

Determination of potential deposition of elemental iron in cardiac, hepatic, or pancreatic tissues for the MRI substudy will be evaluated by MRI T2* of the appropriate anatomical region. For patients in this substudy, MRI imaging will be obtained during screening, during visits at months 6 and 12 of the main study, and at the final visit, approximately 24 months from the initial treatment period visit. Images will be reviewed by a central reading facility imaging core laboratory.

#### Other assessments

Other assessments will include ferumoxytol and iron sucrose exposure, ESA use, and the requirement for red blood cell or whole blood transfusions.

#### Safety assessments

Following each IV administration of study drug during the main study, patients will be required to remain at the clinic for at least 30 min after completion of the administration for monitoring of vital signs and for observation of any immediate adverse reactions to the study drug. At each contact with the patient, the investigator will capture adverse events (AEs) by specific questioning and as appropriate by examination. Safety assessments will include an evaluation of the AE profile over the course of the study and following each treatment period. This includes all AEs, serious AEs, AEs leading to study drug discontinuation, and AEs of special interest, which includes acute hypotension and hypersensitivity reactions including systemic allergic reactions such as anaphylaxis/anaphylactoid reactions and milder symptoms of hypersensitivity. All AEs will be assessed by the investigator and will be classified as serious or nonserious. Serious AEs are defined as any AE that results in death, is life-threatening, results in hospitalization, prolongs hospital stay, results in persistent significant disability or incapacity, a congenital anomaly/birth defect, or an important medical event (i.e., clearly jeopardizes the patient). AEs will also be qualified by intensity (severity), causality, and expectedness. Other safety assessments include vital signs (i.e., blood pressure, heart rate, respiration rate, and body temperature) and physical examination findings (reported as AEs).

### Study endpoints

Endpoints for the main study are shown in Table 3. The primary efficacy endpoint is the mean change in hemoglobin from Baseline to Week 5 for each treatment period. The primary safety evaluation is the assessment of the AE profile over the course of the study. The substudies examine exploratory endpoints. The “oxidative stress” substudy (exploratory safety endpoint) will assess the mean change in biomarkers of oxidative stress/inflammation from Baseline to Week 5 of the initial treatment period in the main study. For the MRI substudy (exploratory endpoint), the primary endpoint is the absolute change in cardiac MRI T2* from Baseline to each follow-up evaluation (i.e., at approximately 6, 12, and 24 months after initial dosing [main study treatment period day 1]) as well as the percentage of an abnormal cardiac T2* occurrence, which is defined as a value of <20 msec. Some secondary endpoints include the proportion of patients developing abnormal cardiac iron deposition (defined as T2* value <20 msec) at any time point, proportion of patients developing cardiac T2* value <10 msec at any time point, change in liver iron concentration, and pancreatic T2*. Measurement of T2* has become the noninvasive standard for evaluation of iron overload and is the most sensitive to iron deposition [19, 20]. Other secondary endpoints for the MRI substudy are summarized in Table 4.

**Table 3:** Ferumoxytol for Anemia of CKD Trial endpoints and safety evaluations: main study and oxidative stress substudy

Endpoints	
Main study	
Primary efficacy endpoint	•Mean change in hemoglobin from Baseline to Week 5 for each treatment period
Secondary efficacy endpoints	•Mean change in transferrin saturation from Baseline to Week 5 for each treatment period •Proportion of patients with an increase in hemoglobin of ≥1.0 g/dL at any time from Baseline to Week 5 for each treatment period
Safety evaluations	•An evaluation of the AE profile over the course of the study and following each course of ferumoxytol or iron sucrose, including serious AEs, AEs leading to study drug discontinuation, all AEs, vital signs (blood pressure, heart rate, respiration rate, and body temperature); physical examination findings; and routine laboratory parameters (hematology, chemistry, and iron panel)
Exploratory endpoints	•Time to subsequent treatment courses of ferumoxytol or iron sucrose •Cumulative intravenous iron exposure per patient over the course of the study •Proportion of patients requiring blood transfusion •Proportion of patients who had a change in ESA dose (≥20% increase or decrease or initiation/cessation of ESA therapy) over the course of the study
Oxidative stress substudy	
Exploratory endpoints	Mean change in the following blood biomarkers from Baseline to Week 5 of the initial treatment period in the main study: •Protein carbonyl content •13-hydroxyoctadecadienoic acid •4-hydroxynonenal •Monocyte chemoattractant protein-1 •Neutrophil gelatinase-associated lipocalin •High-sensitivity interleukin-6

*AE* adverse event, *CKD* chronic kidney disease, *ESA* erythropoiesis-stimulating agent

**Table 4:** Ferumoxytol for Anemia of CKD Trial endpoint and safety evaluations: the MRI substudy

Endpoints	
MRI substudy	
Primary efficacy endpoint	Absolute change in cardiac T2* from Baseline to each follow-up evaluation
Secondary endpoints	•Proportion of patients who develop cardiac T2* value <20 msec at any time point •Proportion of patients who develop cardiac T2* value <10 msec at any time point •Change in liver iron concentration as determined by T2* from Baseline to each follow-up evaluation •Change in pancreatic T2* from Baseline to each follow-up period •Change in mean ferritin, transferrin saturation, liver function test, and thyroid function test values from Baseline to each follow-up evaluation •Change in blood glucose and glycosylated hemoglobin from Baseline to each follow-up evaluation

*CKD* chronic kidney disease, *MRI* magnetic resonance imaging, T2* T2-weighted MRI

### Statistical analysis

The intent-to-treat population will be used for the primary efficacy analysis and includes all patients who had any exposure to ferumoxytol or iron sucrose during the postrandomization period of the study. The evaluable population for the main study includes all patients who meet all inclusion criteria and do not violate any exclusion criteria, receive 2 doses of ferumoxytol (1.02 g) or all 10 doses of iron sucrose at the initial treatment period, and have evaluable data for hemoglobin (primary endpoint) at the treatment period Baseline and Week 5. For the “oxidative stress” substudy, the evaluable population includes all patients who receive 2 doses of ferumoxytol (1.02 g) or all 10 doses of iron sucrose at the initial treatment period and have at least one of the evaluable blood biomarkers of oxidative stress/inflammation at the treatment period Baseline and Week 5. For the MRI substudy, the evaluable population includes all patients who receive study drug and have at least one pair of evaluable Baseline and post-Baseline image data on any of the defined MRI endpoints. The safety population includes all patients who have any exposure to study drug during the study.

A sample size of 240 patients provides approximately 90% power for testing noninferiority of ferumoxytol to iron sucrose, assuming a noninferiority margin of 0.5 g/dL (5.0 g/L), a one-sided alpha of 0.025, and a standard deviation of 1.13 g/dL (11.3 g/L) for the primary endpoint. The noninferiority margin of 0.5 g/dL (5.0 g/L) was selected because it represents 50% of the minimum clinically meaningful change in hemoglobin 1.0 g/dL (10.0 g/L; the expected clinical response with transfusion of 1 unit of packed red blood cells).

## Discussion

FACT will be the longest randomized controlled clinical trial comparing 2 IV iron products in patients with CKD and IDA on hemodialysis. The study will provide efficacy and safety data from a long-term head-to-head trial of IV iron therapy in hemodialysis patients with CKD and IDA, and these results may provide valuable information that addresses knowledge gaps on the use of IV iron in these patients [9]. The study will also address some recognized concerns regarding IV iron therapy, particularly with respect to the impact of IV iron on oxidative stress/inflammation and the longer-term potential for iron overload.

In particular, the FACT study will provide a direct comparison of the efficacy of ferumoxytol versus the recommended dose of IV iron sucrose [21] over 13 months, providing comparative effects of these agents on hematology and iron panel parameters. One limitation of the study was the inability to blind the investigators to study drug due to the different dosing regimens of each drug. However, the primary and secondary efficacy and safety assessments were conducted outside the timeframe for treatment administration, thus limiting the potential for bias. The study will also provide a long-term assessment of the comparative safety of these 2 agents. Furthermore, the substudies will provide a direct comparison of the effect of ferumoxytol and iron sucrose on oxidative stress and the potential for the long-term use of these agents to result in iron deposition in tissue over 2 years. Preclinical data suggest that some IV iron preparations (e.g., iron sucrose and ferric gluconate) induce oxidative stress, whereas ferumoxytol was bioneutral in this regard [22, 23]. Thus, there may be differences in the pro-oxidant effects among IV iron formulations that may be determined by the assessment of oxidative stress associated with various formulations. The study will also provide useful insights into the use of MRI T2* for monitoring iron overload in patients receiving long-term iron supplementation. Thus, FACT fulfills the need for long-term comparative head-to-head studies of IV iron in hemodialysis patients with CKD and IDA, providing an opportunity to assess multiple aspects of safety and efficacy with ferumoxytol in the longest evaluation of 2 IV iron products to date. Recruitment to the study has ended, and completion of the main study is expected soon.

## Abbreviations

AE: Adverse event
CAPD: Continuous ambulatory peritoneal dialysis
CKD: Chronic kidney disease
ESA: Erythropoiesis-stimulating agent
Hb: Hemoglobin
HD: Hemodialysis
ICF: Informed consent form
IDA: Iron deficiency anemia
IV: Intravenous
MRI T2*: T2-weighted magnetic resonance imaging
NDD: Non-dialysis dependent
PD: Peritoneal dialysis
PI: Prescribing information
rHuEPO: Recombinant human erythropoietin
TP: Treatment period
TSAT: Transferrin saturation
