# Correction to: The Ferumoxytol for Anemia of CKD Trial (FACT)—a randomized controlled trial of repeated doses of ferumoxytol or iron sucrose in patients on hemodialysis: background and rationale

**DOI:** 10.1186/s12882-018-0899-0

**Published:** 2018-04-26

**Authors:** Iain C. Macdougall, Naomi V. Dahl, Kristine Bernard, Zhu Li, Alka Batycky, William E. Strauss

**Affiliations:** 1 Department of Renal Medicine, King’s College Hospital, Denmark Hill, London, UK; 2 AMAG Pharmaceuticals, Inc., 1100 Winter Street, Waltham, MA 02451 USA

## Correction

Following publication of the original article [1], the authors reported that one of the authors’ name is spelled incorrectly. In this Correction the incorrect and correct author name are shown. The original publication of this article has been corrected.

Originally the author name has been published as:

- Alka Batyky

The correct author name is:

- Alka Batycky
